# Preoperative computed tomography-based tumoral radiomic features prediction for overall survival in resectable non-small cell lung cancer

**DOI:** 10.3389/fonc.2023.1131816

**Published:** 2023-05-03

**Authors:** Bo Peng, Kaiyu Wang, Ran Xu, Congying Guo, Tong Lu, Yongchao Li, Yiqiao Wang, Chenghao Wang, Xiaoyan Chang, Zhiping Shen, Jiaxin Shi, Chengyu Xu, Linyou Zhang

**Affiliations:** ^1^ Department of Thoracic Surgery, The Second Affiliated Hospital of Harbin Medical University, Harbin, China; ^2^ Department of Radiology, The Second Affiliated Hospital of Harbin Medical University, Harbin, China

**Keywords:** computed tomography, radiomics, non-small cell lung cancer, prognosis, LASSO

## Abstract

**Objectives:**

The purpose of this study was to evaluate whether preoperative radiomics features could meliorate risk stratification for the overall survival (OS) of non-small cell lung cancer (NSCLC) patients.

**Methods:**

After rigorous screening, the 208 NSCLC patients without any pre-operative adjuvant therapy were eventually enrolled. We segmented the 3D volume of interest (VOI) based on malignant lesion of computed tomography (CT) imaging and extracted 1542 radiomics features. Interclass correlation coefficients (ICC) and LASSO Cox regression analysis were utilized to perform feature selection and radiomics model building. In the model evaluation phase, we carried out stratified analysis, receiver operating characteristic (ROC) curve, concordance index (C-index), and decision curve analysis (DCA). In addition, integrating the clinicopathological trait and radiomics score, we developed a nomogram to predict the OS at 1 year, 2 years, and 3 years, respectively.

**Results:**

Six radiomics features, including gradient_glcm_InverseVariance, logarithm_firstorder_Median, logarithm_firstorder_RobustMeanAbsoluteDeviation, square_gldm_LargeDependenceEmphasis, wavelet_HLL_firstorder_Kurtosis, and wavelet_LLL_firstorder_Maximum, were selected to construct the radiomics signature, whose areas under the curve (AUCs) for 3-year prediction reached 0.857 in the training set (n=146) and 0.871 in the testing set (n=62). The results of multivariate analysis revealed that the radiomics score, radiological sign, and N stage were independent prognostic factors in NSCLC. Moreover, compared with clinical factors and the separate radiomics model, the established nomogram exhibited a better performance in predicting 3-year OS.

**Conclusions:**

Our radiomics model may provide a promising non-invasive approach for preoperative risk stratification and personalized postoperative surveillance for resectable NSCLC patients.

## Introduction

Non-small cell lung cancer (NSCLC), the most essential subtype of lung cancer, represents a prevalent malignant tumor with an unsatisfactory prognosis (1). In recent years, an expanding availabilities of targeted therapy and immune checkpoint inhibitors (ICIs), has been approved for lung cancer and improved the long-term prognosis of NSCLC patients. However, due to the secondary mutations and low response rate to ICIs, only a limited number of patients can benefit from those therapeutic approaches (2, 3). Thus, precision diagnose and comprehensive prognostic evaluation are essential steps when dealing with resectable NSCLC patients, in order to select the most appropriate treatment. The tumor node metastasis (TNM) staging system is still a classic evaluation approach which can assist in adjuvant therapy choices and predict the outcome of NSCLC (4). Nonetheless, patients with the same TNM stage typically manifest different clinical outcomes, which is largely attributed to tumor heterogeneity and anatomical factors (5). With the advent of multi-omics, evaluation of prognostic features based on multidisciplinary methods make personalized medicine possible.

Medical imaging has been long regarded as standard procedure for early screening, treatment decision-making, and postoperative surveillance of cancer patients. Computed tomography (CT) imaging, commonly stored in the form of Digital Imaging and Communications in Medicine (DICOM), can be conveniently obtained and utilized for quantitative assessment. Over the last decade, radiomics has emerged as a hot research field that provides massive high-dimensional feature space derived from raw imaging data by automatically high-throughput algorithm (6). There is growing evidence that quantitative parameters and features mined from functional and morphological images offer a new perspective for tumor phenotypes and microenvironment, which also have a significant complementary interrelation with other omics approaches such as genomics, hematology, and proteomics (7–9). Radiomics analyses based on intratumoral and peritumoral regions have been extensively used for exploring underlying biological process, predicting pathological characteristics, evaluating the drug treatment response, and assisting therapeutic decision-making in several human carcinomas (10–14).

As a novelly emerging tool, radiomics provides a new direction of exploring intratumor heterogeneity and predictive markers using a noninvasive evaluation in lung cancer. Using three machine-learning (ML) classifiers derived from radiomics, Liu et al. estimated the benefit from Nivolumab treatment and progression probability in patients with stage IIIB/IV NSCLC (15). In terms of histological subtypes, an automatic deep-learning radiomics model showed satisfactory performance to distinguish lung squamous cell carcinoma (LUSC), lung adenocarcinoma (LUAD), and small cell lung cancer (SCLC) on CT images (16). However, there are limited studies investigating the contingent value of radiomics in improving prognosis stratification of resectable NSCLC patients.

Accordingly, the current study aimed to analyze the association of radiomics features with 3-year overall survival (OS) in enrolled patient cohort. Furthermore, the integration of radiomics model and clinicopathological traits was conducted to establish a comprehensive nomogram, which strengthen the prediction ability and may provide assistance in improving follow-up plans and individualized treatment in clinical practice.

## Materials and methods

### Patient data and study design

**Figure 1:**
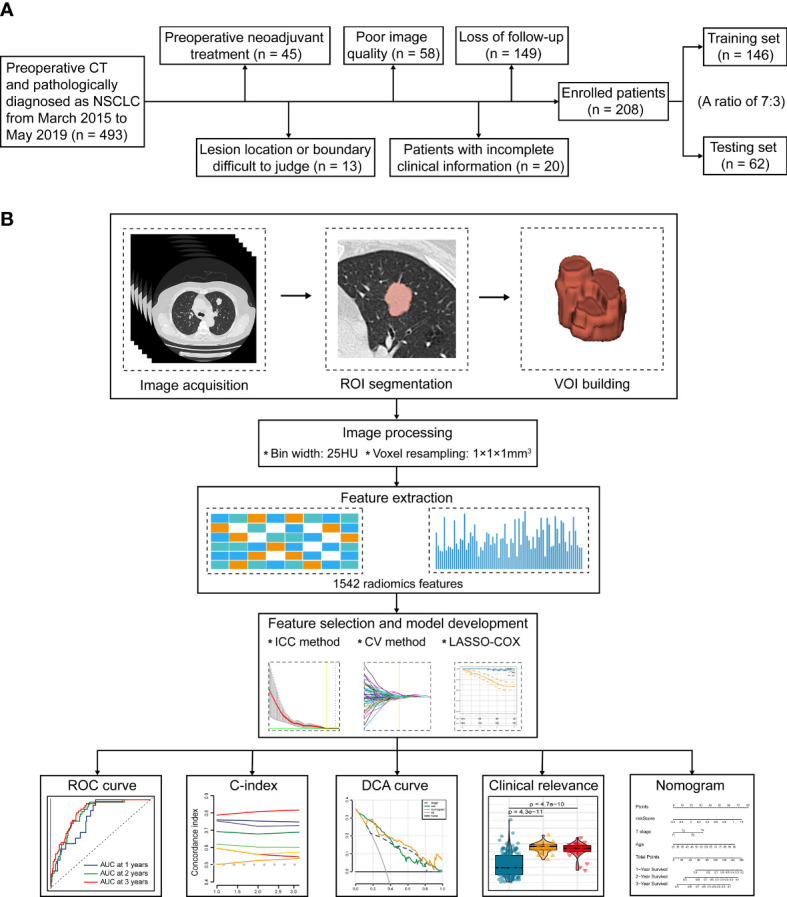
The flowchart of this study. **(A)** Patient selection and exclusion criteria of this work. **(B)** Tumor segmentation, radiomics feature extraction, feature selection, and radiomics model construction.

This study was approved by the Ethics and Scientific Committees of the Second Affiliated Hospital of Harbin Medical University (Approval Number: KY2022-144), due to the retrospective nature of this study, written informed consent for CT images was waived. The study design is illustrated in **Figure 1**.

493 consecutive NSCLC patients who underwent radical surgery (segmental resection, wedge resection, and lobectomy) at our institute from March 2015 to May 2019 were preliminarily included. All patients fulfilled the following inclusion criteria: (1) CT performed within 2 weeks before surgery; (2) Available CT images stored in DICOM format; and (3) Primary NSCLC confirmed by histopathology. The subsequent patient selection and exclusion criteria were visualized in **Figure 1A**. Consequently, we finally recruited 208 consecutive patients, including 167 cases with LUAD, 35 cases with LUSC, 3 cases with large cell carcinoma (LCC), 2 cases with adenosquamous carcinoma (ASC), and 1 case with carcinoid. According to the random allocation scheme and a ratio of 7:3, all patients were separated into two individual cohorts: 146 for training and 62 for testing. Patient grouping and corresponding clinicopathological traits are recapitulated in **Table 1**.

**Table 1:** Patient grouping and corresponding clinicopathological traits.

Covariates		Training set (n=146)	Testing set (n=62)	Total set (n=208)	P-value
Age, no (%)	≤ 65	99(67.81)	48(77.42)	147(70.67)	0.220
	> 65	47(32.19)	14(22.58)	61(29.33)	
Gender, no (%)	Female	77(52.74)	35(56.45)	112(53.85)	0.735
	Male	69(47.26)	27(43.55)	96(46.15)	
Smoking history, no (%)	No	89(60.96)	41(66.13)	130(62.50)	0.584
	Yes	57(39.04)	21(33.87)	78(37.50)	
T stage, no (%)	T1-2	131(89.73)	56(90.32)	187(89.90)	1.000
	T3-4	15(10.27)	6(9.68)	21(10.10)	
N stage, no (%)	N0	115(78.77)	48(77.42)	163(78.37)	0.975
	N1-2	31(21.23)	14(22.58)	45(21.63)	
Pathologic TNM stage, no (%)	Stage I	101(69.18)	46(74.19)	147(70.67)	0.754
	Stage II	21(14.38)	7(11.29)	28(13.46)	
	Stage III	24(16.44)	9(14.52)	33(15.87)	
Lateral location, no (%)	Left	60(41.10)	30(48.39)	90(43.27)	0.413
	Right	86(58.90)	32(51.61)	118(56.73)	
Lobe location, no (%)	Mid-lower	65(44.52)	32(51.61)	97(46.63)	0.432
	Upper	81(55.48)	30(48.39)	111(53.37)	
Location classification, no (%)	Central	29(19.86)	7(11.29)	36(17.31)	0.196
	Peripheral	117(80.14)	55(88.71)	172(82.69)	
Max diameter, no (%)	≤3cm	112(76.71)	49(79.03)	161(77.40)	0.854
	>3cm	34(23.29)	13(20.97)	47(22.60)	
Pathological type, no (%)	LUAD	112(76.71)	55(88.71)	167(80.29)	0.138
	LUSC	29(19.86)	6(9.68)	35(16.83)	
	Other	5(3.42)	1(1.61)	6(2.88)	
Histological grade, no (%)	Well	24(16.44)	8(12.9)	32(15.38)	0.644
	Moderate	44(30.14)	15(24.19)	59(28.37)	
	Poor	50(34.25)	26(41.94)	76(36.54)	
	Unknown	28(19.18)	13(20.97)	41(19.71)	
Radiological sign, no (%)	Cavitary appearance	30(20.55)	21(33.87)	51(24.52)	0.091
	Part-solid appearance	40(27.4)	12(19.35)	52(25)	
	Pure ground-glass appearance	16(10.96)	10(16.13)	26(12.5)	
	Pure solid appearance	60(41.1)	19(30.65)	79(37.98)	

LUAD, lung adenocarcinoma; LUSC, lung squamous cell carcinoma.

### Follow-up

After radical surgery, all enrolled subjects were followed up by outpatient review or telephone every 3 months for the first year and every 6 months thereafter. We applied 3-year OS as the primary study endpoint, which is construed as the time between the operation and the date of all-cause death.

### CT image acquisition and pre-processing

Helical CT images of all enrolled subjects were acquired by 64-channel CT (Discovery 750, GE Healthcare, Milwaukee, USA) and 256-channel CT (Revolution CT, GE Healthcare, Waukesha, WI, USA). Detailed scanning parameters were as follows: tube voltage, 120 kV; tube current, 100-250 mAs; slice thickness, 0.625-5 mm; field of view (FOV), 350-400mm; 512 x 512 matrix; and reconstructed slice thickness, 0.625-3mm. Filtered back projection (FBP) and adaptive statistical iterative reconstruction (ASIR) level 40% were utilized to reconstruct all enrolled CT images. A standard kernel was also used in the reconstruction procedure.

Due to the potential differences in specifications caused by distinct reconstruction slice thickness and voxel spacing, we performed image pre-processing before radiomics feature extracting. Specifically, we chose to resample the raw data to a standard voxel spacing of 1x1x1 mm^3^ by near interpolation algorithm (17). Moreover, we adopted 25 HU as the fixed bin width to perform gray level discretization (18).

### Tumor segmentation and radiomics feature extraction

CT images stored in DICOM format were loaded to 3D slicer (Version 4.1.1) software to perform tumor segmentation. As an open source platform, 3D slicer software has superiority in image interactive segmentation and medical raw data processing (19). A thoracic surgeon with 5 years of experience first manually delineated all lesions slice by slice in the lung window of CT images, the volumes of interest (VOIs) were then inspected and revised by an experienced radiologist. They were blinded to the clinical outcome and medical records of enrolled patients, in parallel, intratumoral or tumor-adjacent vessels, bronchi, and air spaces were carefully avoided during delineation of the tumor volume. To improve the reproducibility of radiomics features, the VOIs of 50 randomly selected CT images were repetitively delineated by another experienced thoracic surgeon, which were used for subsequent interclass correlation coefficient (ICC) analysis.

In our study, 1542 radiomics features were extracted from each manually-defined VOI using “pyradiomics” python package, the detailed definitions of which are congruity with Imaging Biomarker Standardization Initiative (IBSI). The extracted radiomics features can be assigned to the following categories: (1) Gray level dependence matrix-based features (GLDM); (2) neighboring gray tone difference matrix-based features (NGTDM); (3) gray level co-occurrence matrix-based features (GLCM); (4) gray level run-length matrix-based features (GLRLM); (5) gray level size zone matrix-based features (GLSZM); (6) first-order statistics features; (7) shape-based features, and (8) transformed features: features extracted from images pre-processed with several built-in filters including laplacian of gaussian (LoG), wavelet, logarithm, square, square root, and gradient. Detailed mathematic definitions and feature explanations can be acquired from the previous literature (20, 21).

### Feature selection and radiomics model construction

To assess the consistency and robustness of extracted radiomics features between the two thoracic surgeons, we calculated the interclass correlation coefficient (ICC) value for each radiomics feature. The radiomics features with ICC values > 0.75 were retained for deeper analysis. Next, we performed data pre-processing using Z-score transformation by “caret” R package. Least absolute shrinkage and selection operator (LASSO) Cox regression analysis were utilized for further feature selection and model construction. In the training set, LASSO with ten-fold cross-validation effectively avoided over-fitting and identified optimal features with nonzero coefficients by applying a constraint on the model hyperparameter (λ). Finally, we obtained a linear combination of optimal radiomics features weighted with the regression coefficients during multiple computing. All the selected features for model construction are detailed in **Table 2**.

**Table 2:** Six selected radiomics features.

	Selected radiomics feature	Radiomics group	Filter associated	Regression coefficient
R1	InverseVariance	GLCM	gradient	0.068
R2	Median	First order	logarithm	0.096
R3	RobustMeanAbsoluteDeviation	First order	logarithm	0.181
R4	LargeDependenceEmphasis	GLDM	square	0.375
R5	Kurtosis	First order	wavelet_HLL	0.016
R6	Maximum	First order	wavelet_LLL	0.269

GLCM, gray level co-occurrence matrix; GLDM, Gray level dependence matrix.

### Radiomics model validation and evaluation

The prognostic efficacy of radiomics model established in the training set was subsequently verified in the testing set and the total enrolled subjects. Patients in these three sets were respectively dichotomized into low- and high-risk groups based on the median radiomics score threshold. To appraise diversities in the overall survival between two cohorts with different radiomics score, R packages survival and survMiner were used to perform Kaplan-Meier survival analysis. We utilized receiver operating characteristic (ROC) curve analysis and calculated the area under the curve (AUC) to access the sensitivity and specificity of the prognostic model. The R packages survminer, survival, and timeROC were employed to enable this process. Moreover, Harrell’s concordance index (C-index) was applied to estimate the predictive accuracy of the signature. AUC and C-index both ranges from 0.5 (poor predictive performance) to 1 (perfect predictive performance).

A nomogram comprising of radiomics score and clinicopathological parameters (age, gender, smoking history, pathological type, lobe location, location classification, lateral location, max diameter, T stage, N stage, and TNM stage) was set up to predict the 1-, 2-, and 3-year OS of resectable NSCLC patients. Correction curve analysis, ROC curve analysis, and C-index were applied to evaluate the predictive performance of the nomogram. In addition, we performed decision curve analysis (DCA) to compare the clinical benefit of the classical TNM stage, established radiomics model, and the nomogram by quantifying the net benefits (22).

### Statistical analyses

All statistical analyses were carried out by R software (version 4.1.2) and Python software (version 3.4.3). The statistically significant threshold was set to *p* value < 0.05 (*p < 0.05, **p < 0.01, ***p < 0.001).

## Results

### Construction of the radiomics model

All enrolled 208 subjects were randomly divided into training set (n=146) and testing set (n=62) with a ratio of 7:3, and there was no statistical difference in terms of clinical features (**Table 1**). The training set was applied to create the prognostic radiomics model, we then used the testing set and the total set to validate the constructed model.

**Figure 2:**
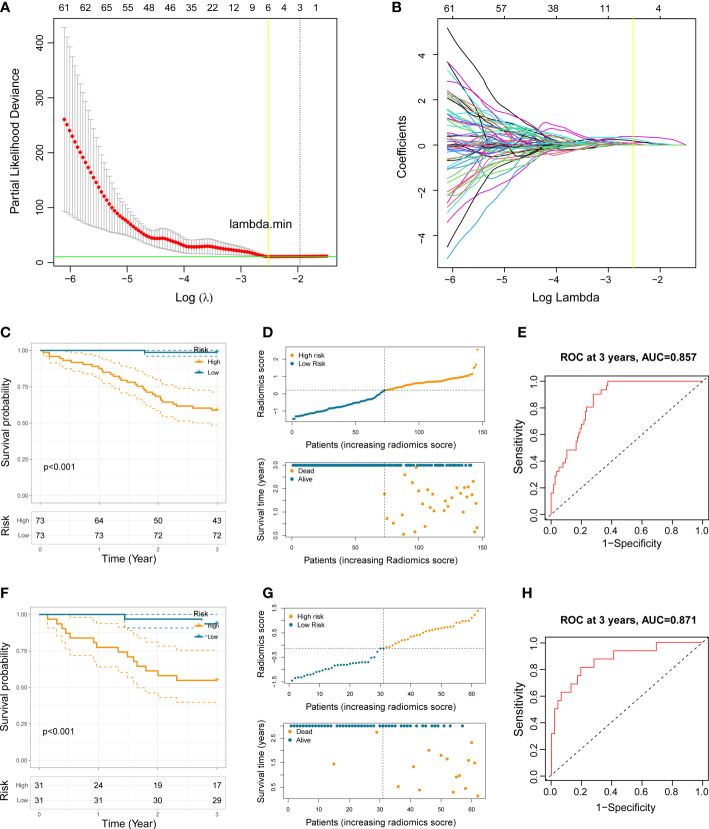
Construction of the prognostic radiomics model. **(A)** Selection of the tuning parameter (log λ) based on minimum criteria in the LASSO analysis. **(B)** LASSO coefficient profiles. **(C, F)** Kaplan-Meier analysis in the training set and the testing set. **(D, G)** The distribution patterns of radiomics score, survival time, and survival status of NSCLC patients in the training set and the testing set. **(E, H)** ROC curves of the radiomics model at 3 years in the training set and the testing set.

According to the 1542 radiomics features extracted by two experienced thoracic surgeons, The ICC analysis indicated good consistency in 1178 (76.39%) radiomics features, moderate consistency in 266 (17.25%) radiomics features, and poor consistency in 98 (6.36%) radiomics features. We ultimately included the 1178 most stable features in the subsequent model construction. LASSO Cox regression analysis is widely used in multiple regression analysis, which not only optimize the selection of characteristics with a deficient correlation and prominent predicted value from high-dimensional data, but also improve the forecast accuracy. We performed tenfold cross-validation to select the minimal penalty term (λ) (**Figures 2A, B**).

Afterwards, we established a prognostic radiomics model implicating six selected features for resectable NSCLC patients. The formula was constructed as follows: Radiomics score = 0.068 × gradient_glcm_InverseVariance + 0.096 × logarithm_firstorder_Median + 0.181 × logarithm_firstorder_RobustMeanAbsoluteDeviation + 0.375 × square_gldm_LargeDependenceEmphasis + 0.016 × wavelet_HLL_firstorder_Kurtosis + 0.269 × wavelet_LLL_firstorder_Maximum.

### Survival analyses in the training set, testing set, and the entire NSCLC set

**Figure 3:**
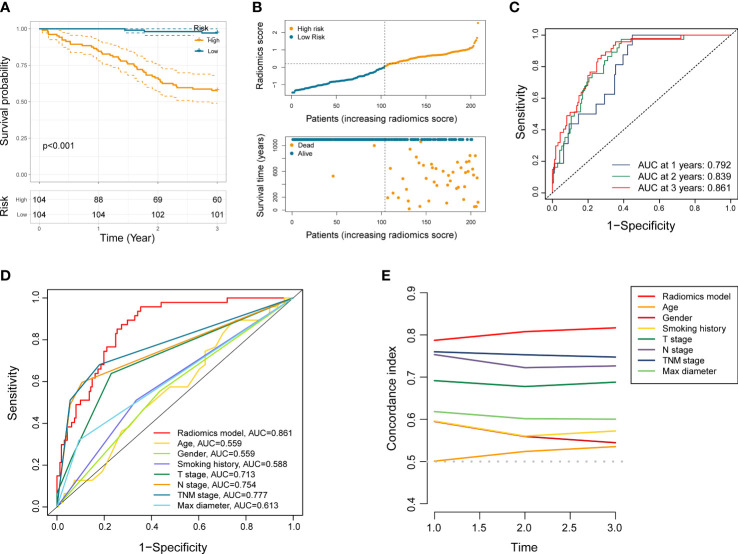
Performance evaluation of the radiomics model. **(A)** Kaplan-Meier analysis in entire NSCLC set. **(B)** The distribution patterns of radiomics score, survival time, and survival status of NSCLC patients in entire NSCLC set. **(C)** ROC analysis of the radiomics model in entire NSCLC set at 1 year, 2 years, and 3 years, respectively. **(D, E)** ROC and C-index curves comparing the radiomics model and other clinical characteristics.

We first dichotomize 146 NSCLC patients of training set into low- and high-risk groups based on the median radiomics score. Kaplan-Meier analysis showed significantly distinct prognoses between the two risk groups (**Figure 2C**). In addition, the distributions of the radiomics score, survival time, and survival status of each NSCLC patient in the training set were shown in **Figure 2D**. To further verify the prognostic performance of the constructed radiomics model, we acquired the radiomics score of each NSCLC patient in the testing set and the total NSCLC set using the radiomics score formula. Employing similar segmentation method, we divided the testing set and the total NSCLC set into subgroups with different risk level. The Kaplan-Meier analysis demonstrated that the patients in the high-risk group had significantly poorer outcome (**Figures 2F**, **3A**). The distribution patterns of the radiomics score, survival time, and survival status of both sets were illustrated in **Figures 2G**, **3B**.

### Performance evaluation of the radiomics model

ROC curve analysis indicated that the AUC of established radiomics model reached 0.857 and 0.871 at 3 years in the training set and the testing set (**Figures 2E, H**). Notably, the AUC of our model in total NSCLC set reached 0.792, 0.839, and 0.861 at 1 year, 2 years, and 3 years, respectively (**Figure 3C**). When compared with other clinicopathological variables in terms of ROC analysis, the radiomics model still had an advantage in evaluating precision and sensitivity (**Figure 3D**). Furthermore, The C-index curve showed promising predictive accuracy of the radiomics model from another dimension (**Figure 3E**).

**Figure 4:**
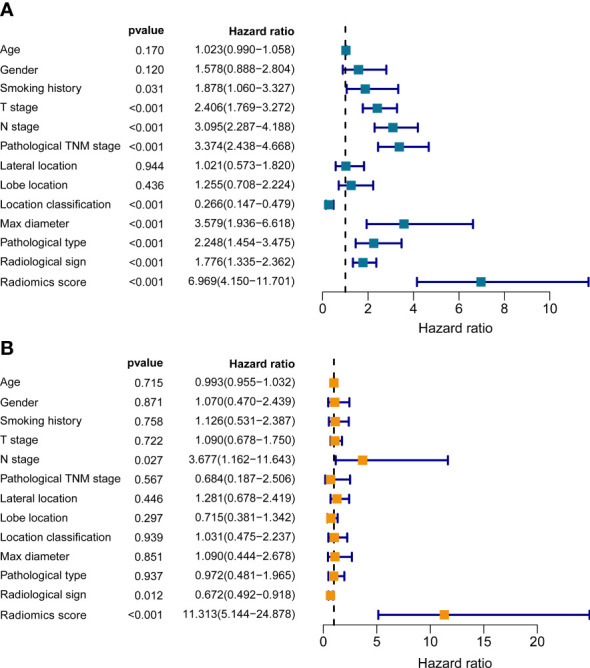
The univariate **(A)** and multivariate **(B)** Cox regression analyses of radiomics score and other clinical traits.

To assess the independent prognostic value of the radiomics model, we incorporated age, gender, smoking history, pathological type, lobe location, location classification, lateral location, max diameter, T stage, N stage, TNM stage, radiological sign, and radiomics score into the univariate and multivariate Cox regression analyses. The results suggested that the established radiomics model, N stage, and radiological sign could act as independent predictors for overall survival in resectable NSCLC patients (**Figure 4**, *p* < 0.001, *p* < 0.05, and *p* < 0.05).

**Figure 5:**
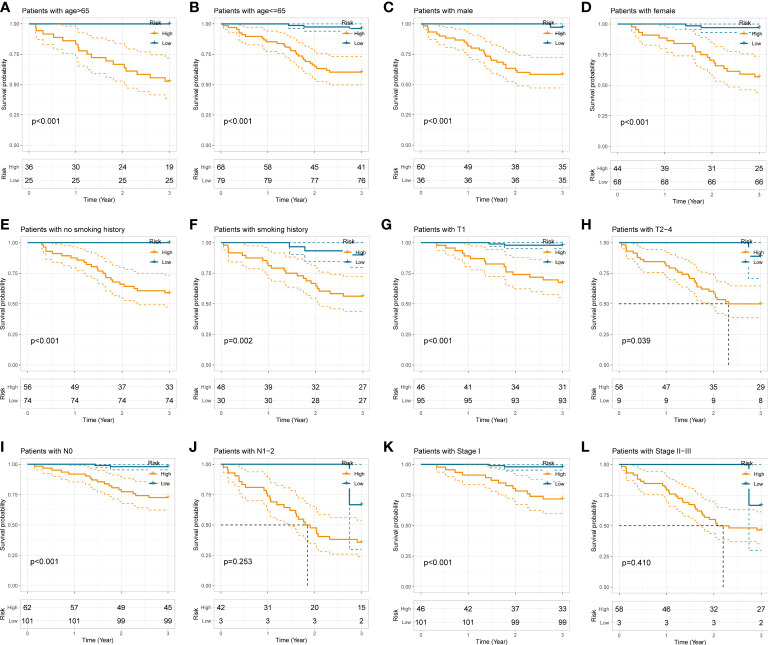
Stratification analyses of the radiomics model in different subgroups stratified by age **(A, B)**, gender **(C, D)**, smoking history **(E, F)**, T stage **(G, H)**, N stage **(I, J)**, and pathological TNM stage **(K, L)**.

To examine the applicability of our radiomics model in subgroups stratified by different clinicopathological traits, the total 208 NSCLC subjects were dichotomized into disparate subgroups by age, gender, smoking history, histological grade, lobe location, location classification, lateral location, max diameter, T stage, N stage, TNM stage, and radiological sign. In the overwhelming majority of subgroups, our radiomics model could accurately distinguish those high-risk patients with worse outcome (**Figure 5** and **Supplementary Figure S1**).

### Correlation between constructed radiomics model and clinicopathological features

**Figure 6:**
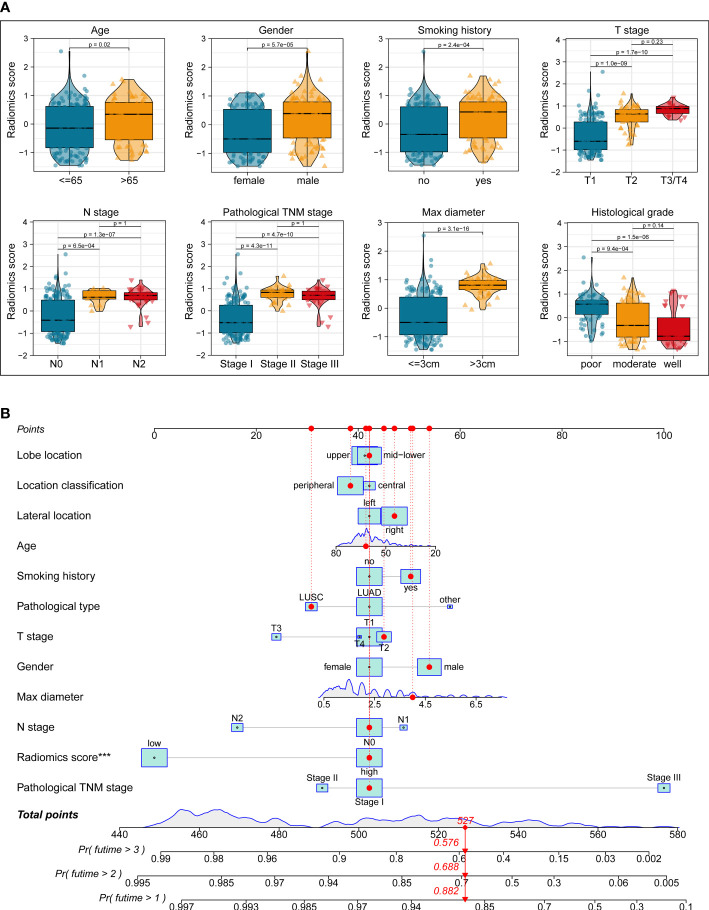
Correlation analyses **(A)** between radiomics score and clinicopathological features. A comprehensive nomogram **(B)** integrating the radiomics model and clinicopathological parameters to predict the 1-, 2-, and 3-year OS of resectable NSCLC patients.

We next performed correlation analysis to dig deeper connections between radiomics score and the clinicopathological traits obtained from the electronic medical record. The results revealed that higher radiomics score with worse survival was significantly associated with age > 65 (*p* < 0.05), male (*p* < 0.001), smoking history (*p* < 0.001), T2-4 (*p* < 0.001), N1-2 (*p* < 0.001), TNM stage II-III (*p* < 0.001), max diameter > 3cm (*p* < 0.001), poor differentiation grade (*p* < 0.001), central-type NSCLC (*p* < 0.001), LUSC and other pathological types (*p* < 0.01), middle and lower lobes (*p* < 0.01), and pure solid appearance (*p* < 0.001), which is similar to previous experiences acquired from clinical practice and may provide additional clues to clinical management of NSCLC (**Figure 6A** and **Supplementary Figure S2**).

### Development and assessment of the prognostic nomogram

**Figure 7:**
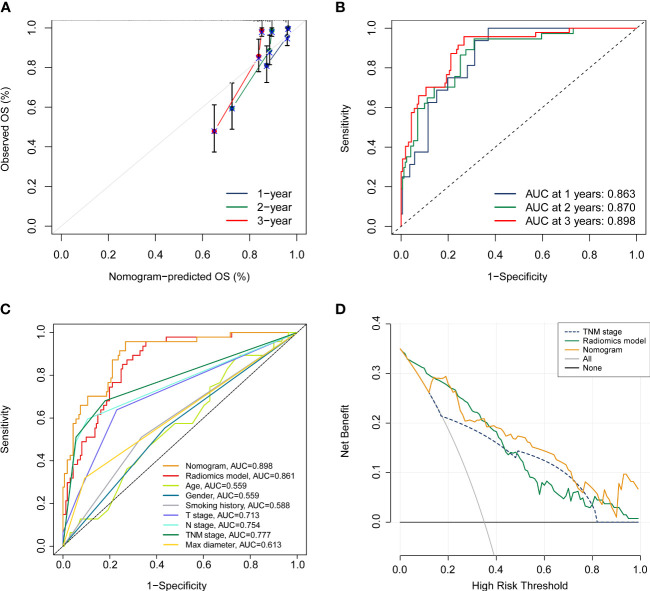
Assessment of the prognostic nomogram. **(A, B)** calibration curve and ROC analyses of the established nomogram at 1 year, 2 years, and 3 years, respectively. **(C)** Comparison of the comprehensive nomogram, radiomics model, and other clinical characteristics by ROC curves. **(D)** DCA curves of the nomogram, radiomics model, and pathological TNM stage.

In order to furnish a comprehensive prognostic tool to predict the survivability of resectable NSCLC patients at 1, 2, and 3 years, we constructed a nomogram integrating the radiomics model and clinicopathological parameters (**Figure 6B**). In terms of predicting OS at 1 year, 2 years, and 3 years, calibration plot displayed that there is a decent consistency between the prediction curve and the ideal curve (**Figure 7A**). The AUC values of the prognostic nomogram were 0.863, 0.870, and 0.898 at 1-, 2−, and 3−year, respectively, which exhibited better performance than radiomics model and other clinicopathological parameters (**Figures 7B, C**). Moreover, the C-index values of the comprehensive nomogram and established radiomics model reached 0.854 and 0.814 at 3 years, respectively. Clinical utility is commonly utilized to measure the practical clinical value of prognostic models. Subsequent DCA curves revealed that the appropriate combination of radiomics model and the comprehensive nomogram may bring significantly more benefit than TNM staging system in clinical work (**Figure 7D**).

## Discussion

In this retrospective study, we explored the potential value of radiomics features in predicting 3-year OS for resectable NSCLC patients varied from pathologic stage IA to stage IIIA. The established radiomics model exhibited good prediction accuracy with an AUC of 0.857 in the training set (n = 146) and an AUC of 0.871 in the testing set (n = 62). The stratified analysis indicated that our radiomics model could distinguish those patients with worse prognosis in the vast majority of subgroups. Subsequently, a comprehensive nomogram incorporating radiomics model and clinical parameters further enhanced the prognostic performance of the single radiomics model with an AUC of 0.898. Thus, this radiomics classifier could be an advantageous noninvasive biomarker in the whole-course clinical management of resectable NSCLC patients.

The generic workflow of NSCLC biomarkers comprises various prognostic models reliant on clinical elements including lserum tumor markers, specific gene expression, next-generation sequencing, and circulating tumor DNA (ctDNA) (23–25). For instance, lu et al. constructed the tumor mutation index (TMI) model based on ctDNA sequencing to predict OS and recognize NSCLC patients who may respond well to monotherapy with atezolizumab or docetaxel (26). To improve NSCLC treatment in terms of chemotherapy and immunotherapy, Guo et al. developed a 7-gene predictive signature using qRT-PCR assays based on 337 snap-frozen NSCLC tissues. Patients receiving adjuvant chemotherapy were precisely identified with significantly better disease-specific survival in the predicted benefit group *via* the multi-gene prognostic signature (27). However, the few available predictive models based on quantitative gene expression levels in the clinical practice have usual limitations such as invasive procedures, time-consuming, cost-effectiveness, and some degree of interference to clinical workflow.

In contrast, radiomics have exhibited a bright prospect for prognosis, diagnosis, and treatment response prediction, as well as long-term health surveillance of NSCLC treatment in a non-invasive modality. Several studies focused on exploring potential combined signature based on radiomics features for NSCLC patients at specific pathologic stage. Huang et al. extracted 132 texture features from CT images of early stage NSCLC (stage I or II) and obtained a better performance for disease-free survival (DFS) prediction (C-index = 0.72) when incorporating the radiomics signature into a comprehensive nomogram (28). Xie et al. enrolled 554 candidates with resected stage I LUAD from three multicenter cohorts and further recognized potential subjects who may benefit from adjuvant chemotherapy (29). Moreover, a number of studies mined high-dimensional clues from functional and metabolic images of ^18^F-FDG-PET/CT, aiming to improve clinical decision of epidermal growth factor receptor (EGFR) tyrosine kinase inhibitors (TKIs) or immune checkpoint inhibitors (ICIs) treatments for specific NSCLC patient populations (30–34). In this study, we restricted the entrance criteria to preoperative CT scans, since CT scans serve as the major approaches for lung cancer screening and whole-course monitoring in a real-world clinical environment, especially in relatively early stages of NSCLC.

Six screened radiomics features used in our prediction model consisted of four first-order-based features (Maximum, Median, Kurtosis, and RobustMeanAbsoluteDeviation), one GLDM-based feature (LargeDependenceEmphasis), and one GLCM-based feature (InverseVariance), which were extracted from CT images pre-processed with built-in filters including wavelet_HLL, wavelet_LLL, logarithm, square, and gradient. First-order statistics features are generally acquired by measuring the gray values of region of interest (ROI) cropping, which reflects the intratumoral distribution of grayscale intensity (20). Previous literature indicated that two First-order statistics features may serve as radiomics predictors for identifying invasive phenotype of solitary pulmonary nodule (35). Moreover, GLDM-based features manifest the intrinsic grayscale associations of central voxel with neighboring voxels, which may be a reflection of heterogeneity and homogeneity of tumors. Padmakumari et al. demonstrated that LargeDependenceEmphasis, a radiomics feature from GLDM, can exhibit robust performance in discriminating lung cancer from tuberculosis with an AUC of 0.92 (36). GLCM describes the integrated information about spatial correlation characteristics of pixel pairs in terms of the pattern of grayscale arrangement, direction, distance, and gray value (37). Notably, textural features derived from GLCM have been demonstrated to have pathological association and can be applied to the diagnosis of malignant lesion in breast cancer (38). Granata et al. identified Correlation from GLCM as a reliable predictor for recognizing tumor recurrence in colorectal liver metastases patients (39).

In our study, we identified the established radiomics model, N stage, and radiological sign as independent predicting indicators for OS in in resectable NSCLC patients. Indeed, several studies have confirmed the accuracy and reliability of radiomics signature in predicting prognosis in NSCLC (40–42). Yang et al. incorporated the radiomics signatures and four clinicopathological features (N stage, T stage, age, and sex) to construct a comprehensive nomogram for survival prediction in NSCLC patients at stage I/II, the performance of which was measured by a C-index of 0.710 (41). The radiomics model and corresponding nomogram in this study showed more satisfactory performance with C-index values of 0.814 and 0.854, respectively. Subsequent DCA and calibration analyses further supported their clinical utility. In the current clinical practice, adjuvant chemotherapy after surgery is not recommended for patients with pathologic stage IA and there is a controversial debate regarding its potential benefit for stage IB (43). In the model evaluation section, our radiomics model seemed to be more robust than traditional TNM staging system. In stratified analyses, the established radiomics model could dichotomize participants with pathologic stage I into high- and low-risk groups by Kaplan-Meier method, which would be helpful to performed personalized treatment interventions on these high-risk patients with worse prognosis.

Some limitations of this study have to be acknowledged. First, due to the relatively small sample size from single center and the retrospective nature, potential selection bias may obstruct the robustness and generalizability of our radiomics model. Therefore, it is necessary to recruit more subjects and perform multicentric external verification in future research. Second, the 3-year follow-up period of included subjects was relatively short, we will conduct the remaining follow-up until 5 years in the next work. Third, we delineated all lesions manually, which was laborious and time-consuming. Automatic delineation based on deep learning method is worth further study to improve the workflow of radiomics in busy clinical practice.

In summary, the current study proposed a novel non-invasive approach based on preoperative CT scans that can predict OS in patients with NSCLC after radical surgery, which may provide clues to help clinicians improve clinical decisions and guide personalized treatment. However, further external validation is warranted before its widespread application in clinical practice.

## Data availability statement

The raw data supporting the conclusions of this article will be made available by the authors, without undue reservation.

## Ethics statement

The studies involving human participants were reviewed and approved by The Ethics and Scientific Committees of the Second Affiliated Hospital of Harbin Medical University. Written informed consent for participation was not required for this study in accordance with the national legislation and the institutional requirements.

## Author contributions

BP conceived the study, conducted radiomics analysis, and wrote the preliminary draft. KW and RX participated in the radiomics analysis and revised the manuscript. CG, TL, YL, CW, and XC contributed to study design and data preprocess. YW contributed to tumor segmentation and provided professional suggestions for statistical analysis. ZS, JS, and CX contributed to data collection. The manuscript was revised and approved by LZ. All authors contributed to the article and approved the submitted version.
