# Supplementary material for: Preoperative computed tomography-based tumoral radiomic features prediction for overall survival in resectable non-small cell lung cancer
Source: Front Oncol. 2023 May 3;13:1131816. doi: 10.3389/fonc.2023.1131816 (PMC10189057; doi:10.3389/fonc.2023.1131816)

**Supplementary Material**

**Supplementary Figure S1.** Stratification analyses of the radiomics model in different subgroups stratified by lateral location **(A, B)**, lobe location **(C, D)**, location classification **(E, F)**, max diameter **(G, H)**, histological grade **(I, J)**, and radiological sign **(K, L, M)**.


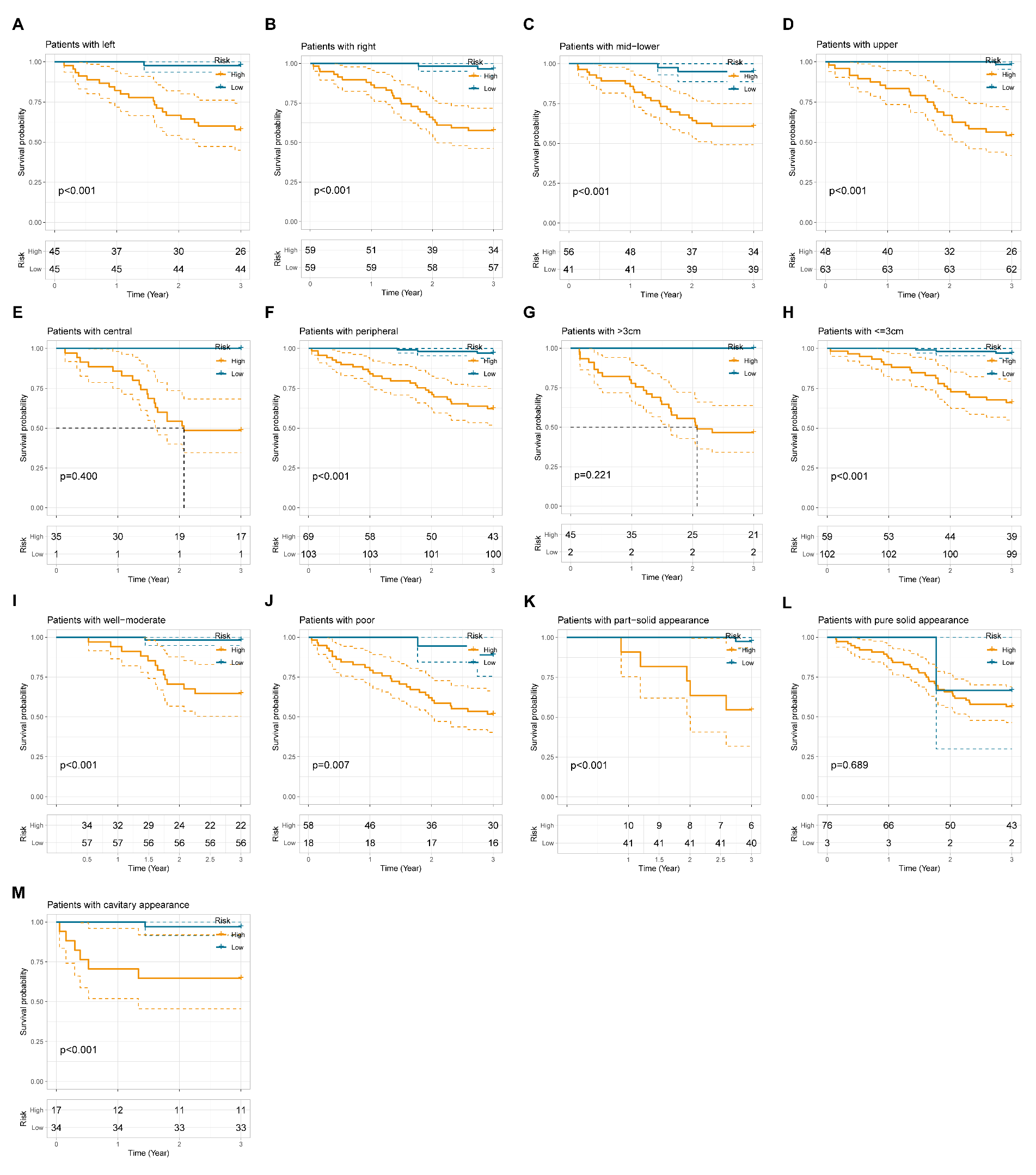


**Supplementary Figure S2.** Correlation analyses between radiomics score and clinicopathological features including location classification **(A)**, pathological type **(B)**, lobe location **(C)**, lateral location **(D)**, and radiological sign **(E)**.


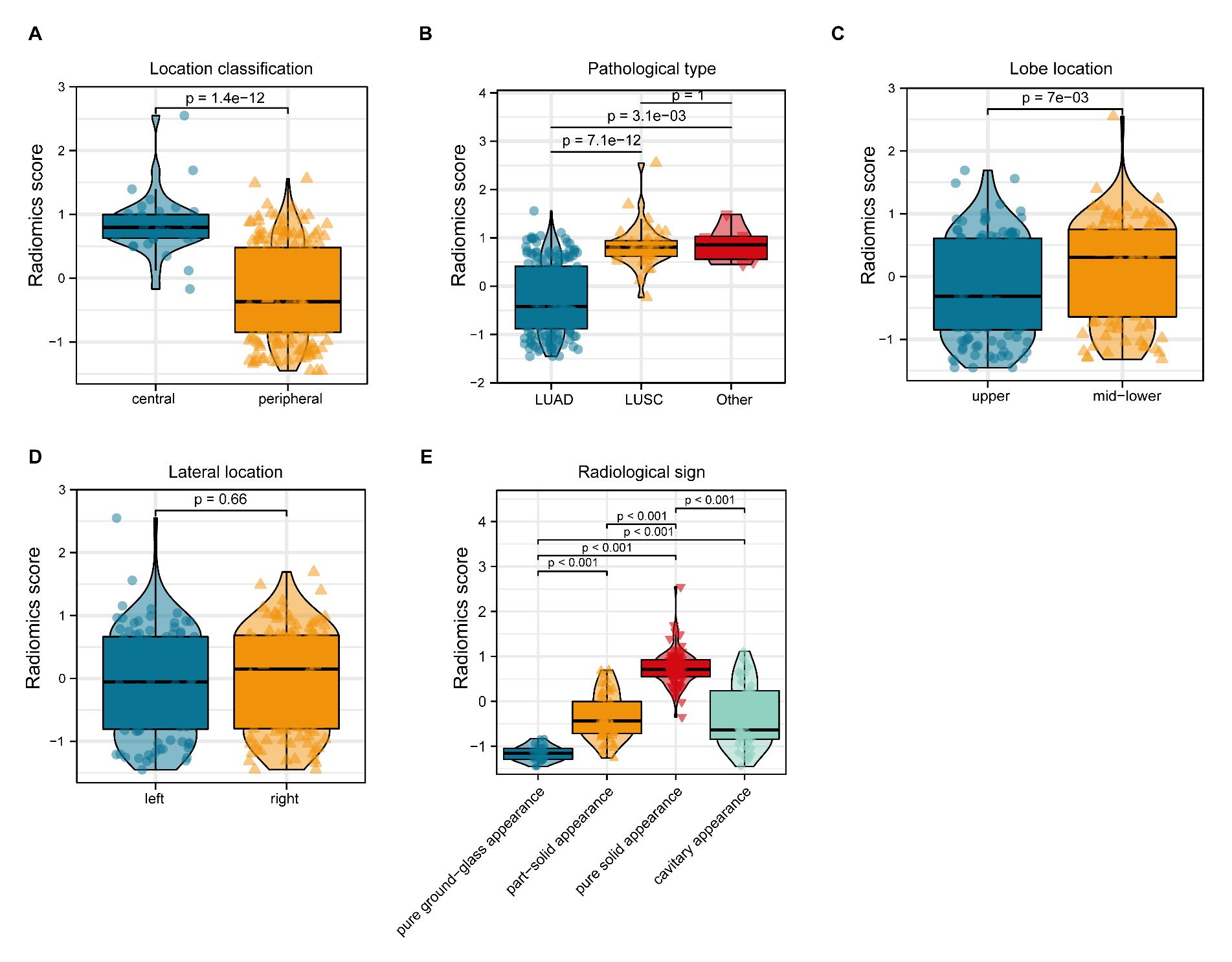

Supplement: Supplementary file 1 [file DataSheet_1.docx]
